# Author Correction: Transgenerational effects of early life stress on the fecal microbiota in mice

**DOI:** 10.1038/s42003-024-06445-6

**Published:** 2024-06-18

**Authors:** Nize Otaru, Lola Kourouma, Benoit Pugin, Florentin Constancias, Christian Braegger, Isabelle M. Mansuy, Christophe Lacroix

**Affiliations:** 1 Nutrition Research Unit, University Children’s Hospital Zürich, Zürich, Switzerland; 2 Department of Health Sciences and Technology, Laboratory of Food Biotechnology, ETH Zürich, Zürich, Switzerland; 3 Department of Health Science and Technology of the ETH Zurich, Laboratory of Neuroepigenetics, Brain Research Institute, Medical Faculty of the University of Zurich, and Institute for Neuroscience, Zurich, Switzerland; 4 Center for Neuroscience Zürich, ETH and University Zürich, Zurich, Switzerland

**Keywords:** Microbiome, Stress and resilience, Microbiota

Correction to: *Communications Biology* 10.1038/s42003-024-06279-2, published online 31 May 2024

The original version of the Article lacked one of the funding sources in the Acknowledgements section. This has now been added and reads as follows: “L.K. received financial support from the Department of Health Science and Technology of ETH Zürich”.

