# Supplementary material for: Transgenerational effects of early life stress on the fecal microbiota in mice
Source: Commun Biol. 2024 May 31;7:670. doi: 10.1038/s42003-024-06279-2 (PMC11143345; doi:10.1038/s42003-024-06279-2)
Supplement: Supplementary file 2 — Supplementary information [file 42003_2024_6279_MOESM2_ESM.pdf]

Supplementary File

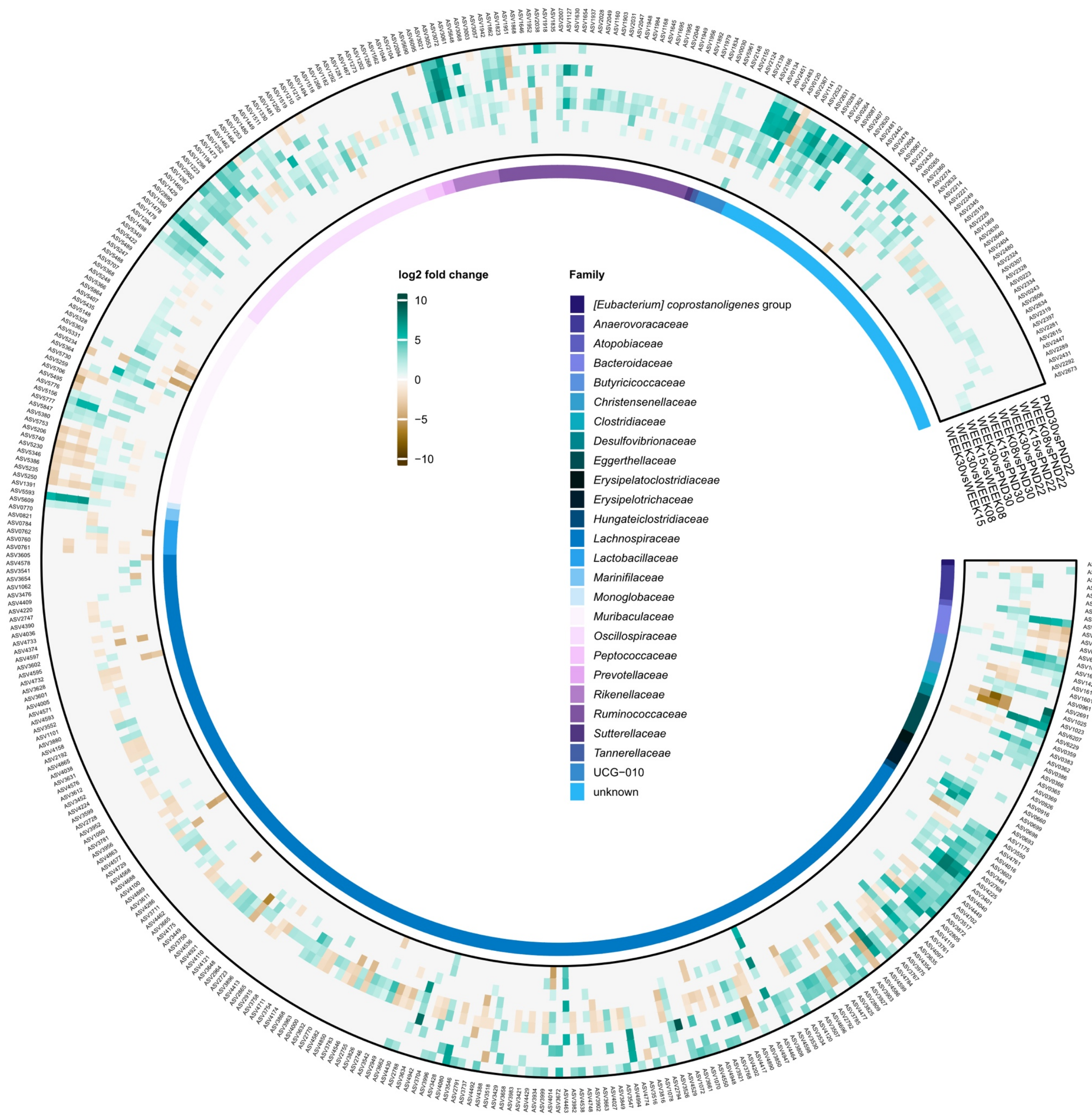

Supplementary Figure 1: Effect of age on relative abundance of ASVs of F1 control mice fecal microbiota. Log_2 fold-change in specific ASVs significantly ($p < 0.05$, FDR-adjusted) decreased or increased across life span. Visualization of pairwise comparison of different ages. Taxonomic information is indicated at family level. Significances were calculated using log_2 transformed abundance counts and generalized mixed effect models with FDR correction. PND: postnatal day

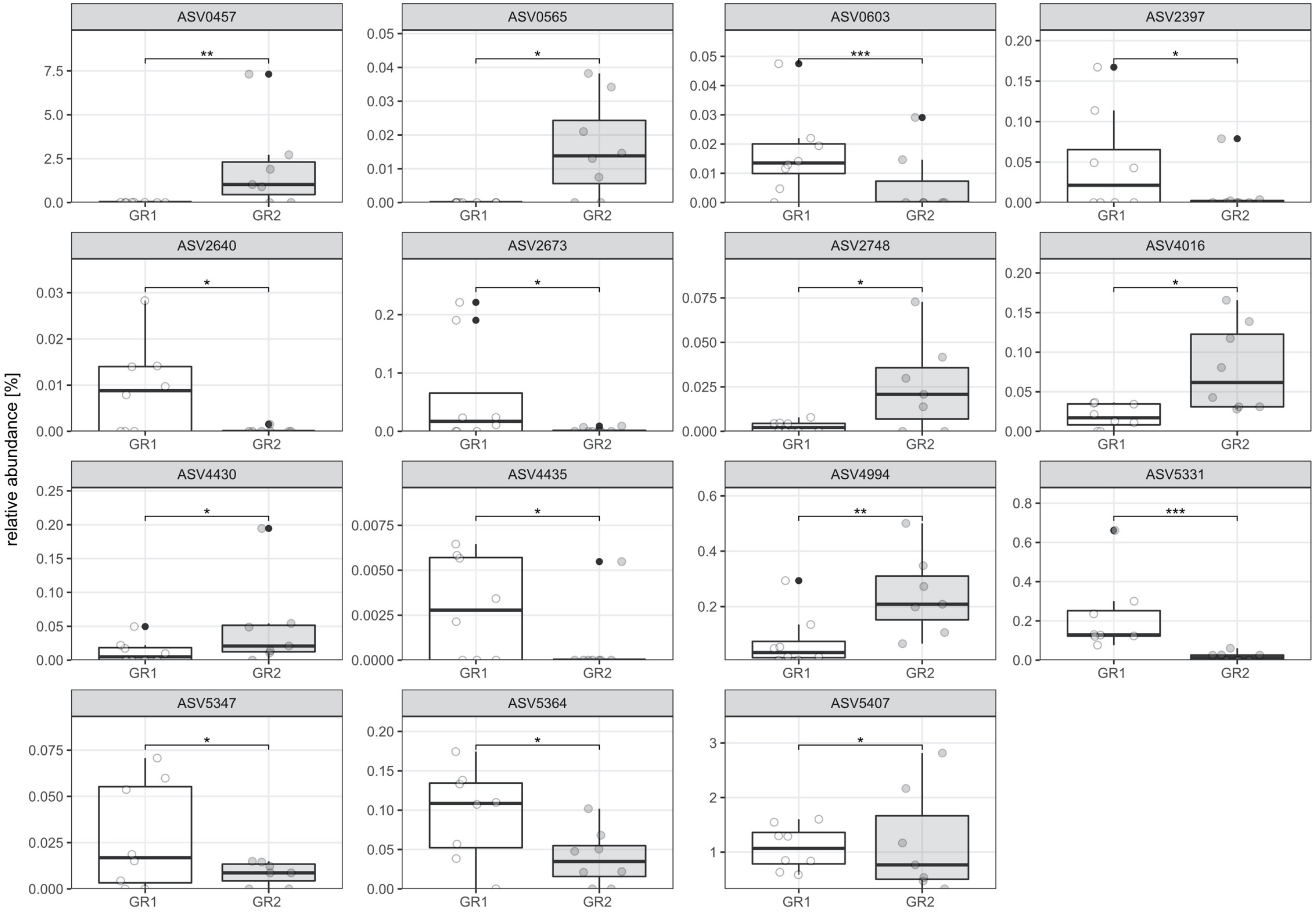

Supplementary Figure 2: Effect of temporal succession of behavioral phenotyping and breeding on relative abundance of ASVs in 30-week-old control mice fecal microbiota. Comparison of phenotyping group 1 (GR1; behavioral phenotyping before breeding) *versus* group 2 (GR2; breeding before behavioral phenotyping) microbiota**.** Significantly differentially abundant ASVs are displayed. Boxplot with box elements showing upper and lower quantile and median. Whiskers extend from the hinge to ± 1.5 times the interquartile range or the highest/lowest value. Outliers are indicated as black points, while white and grey points indicate individual microbiota. Significances were calculated using generalized mixed effect models with FDR correction. * $p < 0.05$; ** $p < 0.01$; *** $p < 0.001$

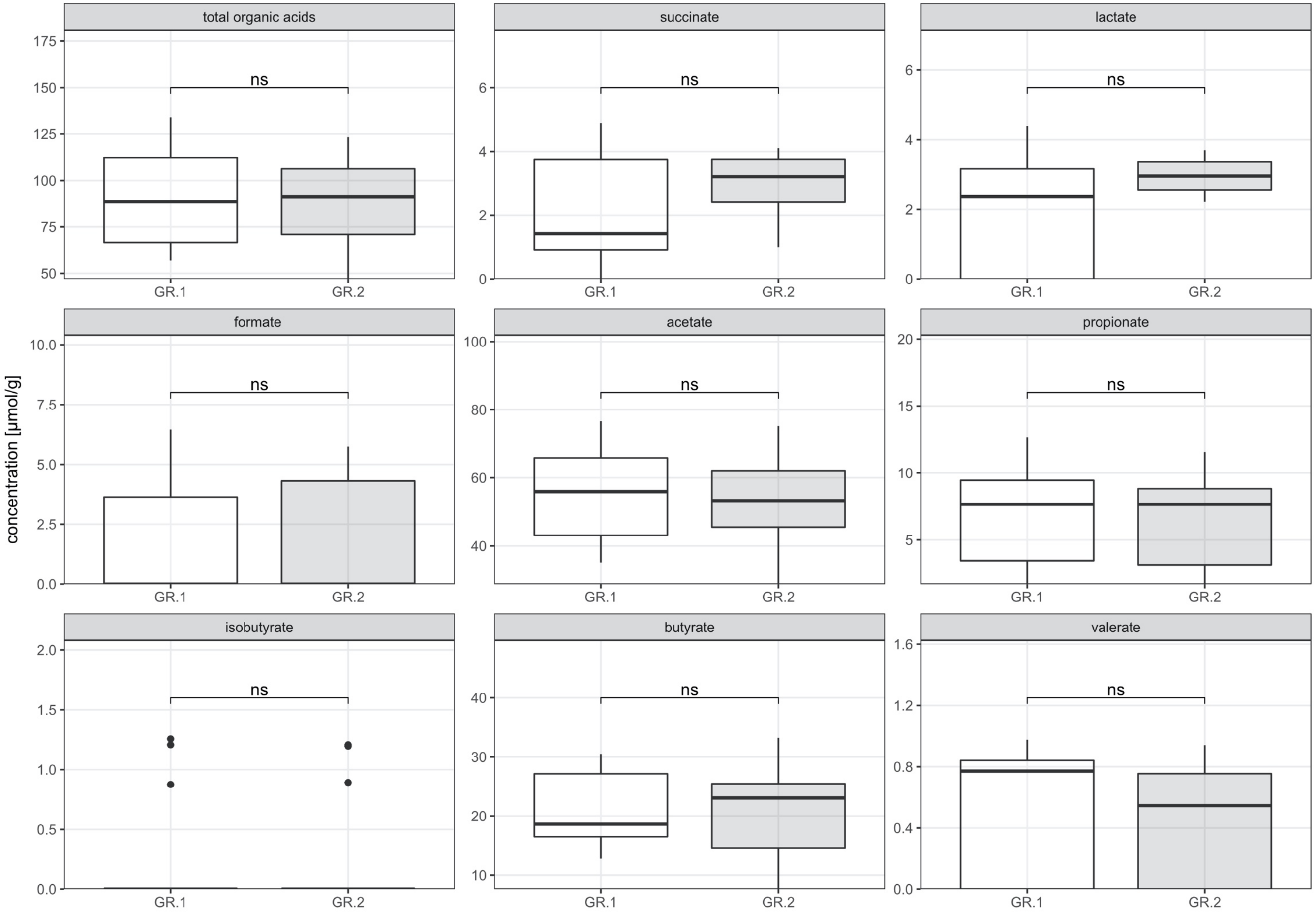

Supplementary Figure 3: Effect of temporal succession of behavioral phenotyping and breeding on cecal total and specific organic acid concentrations of 30-week-old control mice. Comparison of phenotyping group 1 (GR.1; behavioral phenotyping before breeding) *versus* group 2 (GR.2; breeding before behavioral phenotyping) microbiota is displayed**.** Metabolite concentration is depicted per gram cecal wet weight. Boxplot with box elements showing upper and lower quantile and median. Whiskers extend from the hinge to ± 1.5 times the interquartile range or the highest/lowest value. Outliers are indicated as black points. Significances were calculated using generalized mixed effect models with FDR correction. ns: not significant

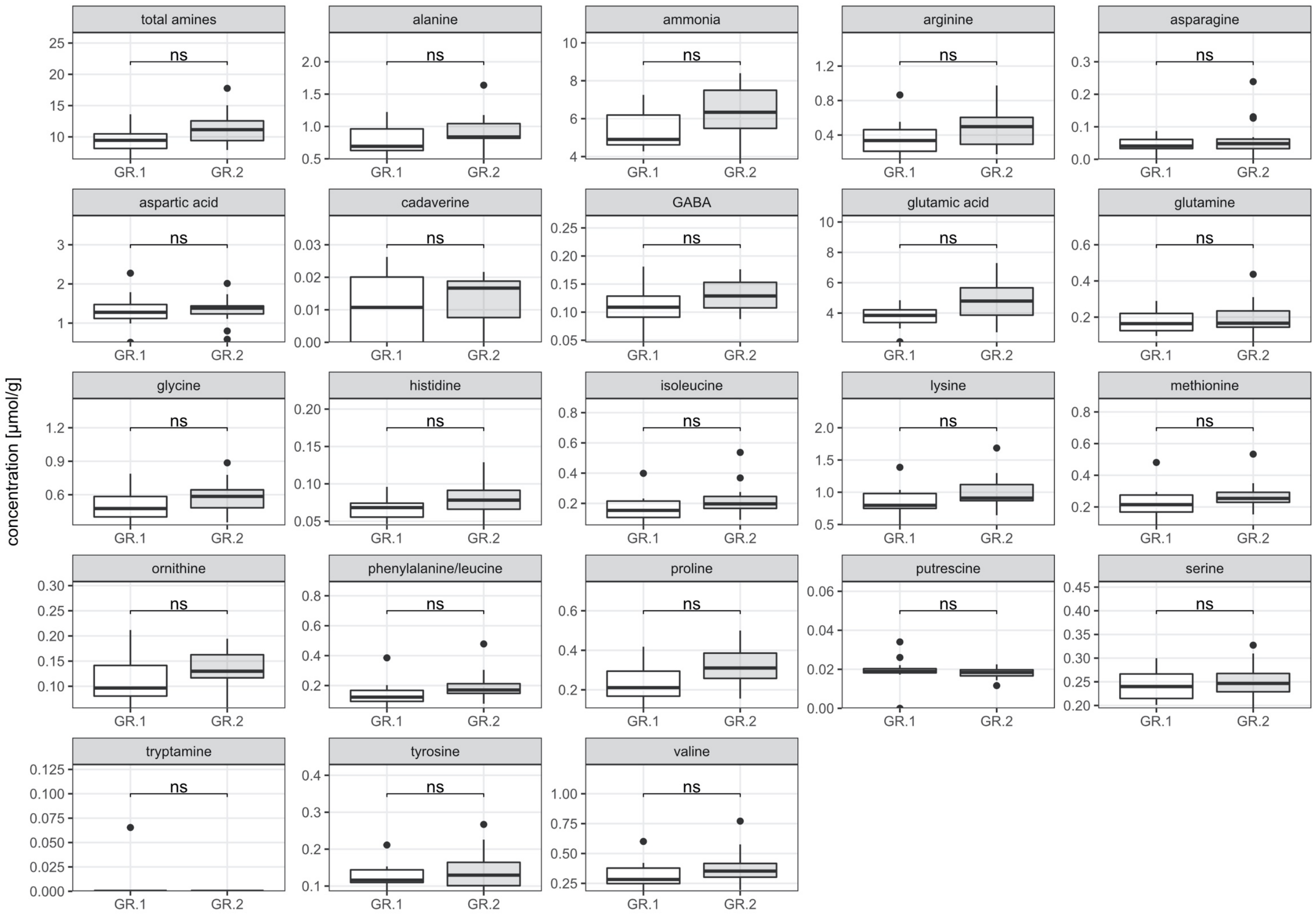

Supplementary Figure 4: Effect of temporal succession of behavioral phenotyping and breeding on cecal amine, amino acid, and ammonia concentrations of 30-week-old control mice. Comparison of phenotyping group 1 (GR.1; behavioral phenotyping before breeding) *versus* group 2 (GR.2; breeding before behavioral phenotyping) microbiota is displayed**.** Metabolite concentration is depicted per gram cecal wet weight. Boxplot with box elements showing upper and lower quantile and median. Whiskers extend from the hinge to ± 1.5 times the interquartile range or the highest/lowest value. Outliers are indicated as black points. Significances were calculated using generalized mixed effect models with FDR correction. ns: not significant

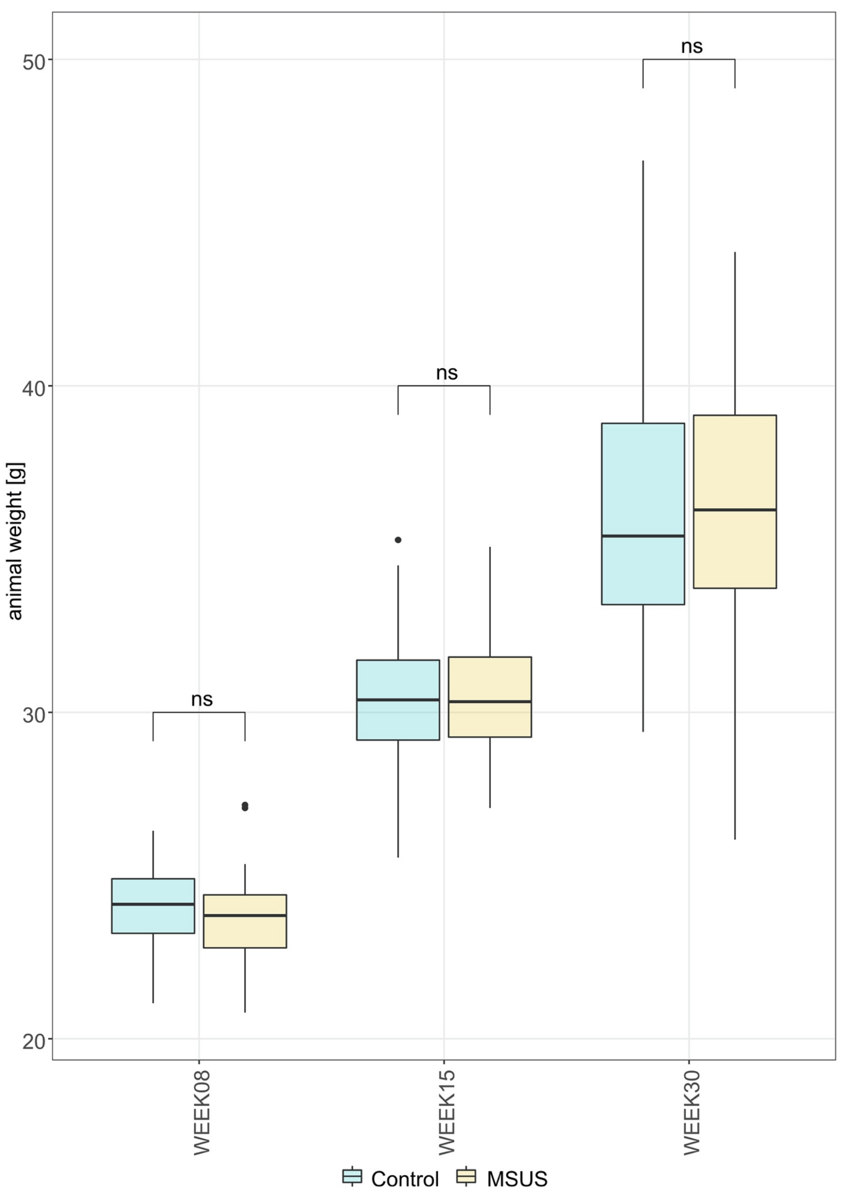

Supplementary Figure 5: Effect of MSUS paradigm on animal weight of F1 mice across life span. Comparison of MSUS *versus* control mice. Boxplot with box elements showing upper and lower quantile and median. Whiskers extend from the hinge to ± 1.5 times the interquartile range or the highest/lowest value. Outliers are indicated as black points. Significances were calculated using generalized mixed effect models with FDR correction. ns: not significant

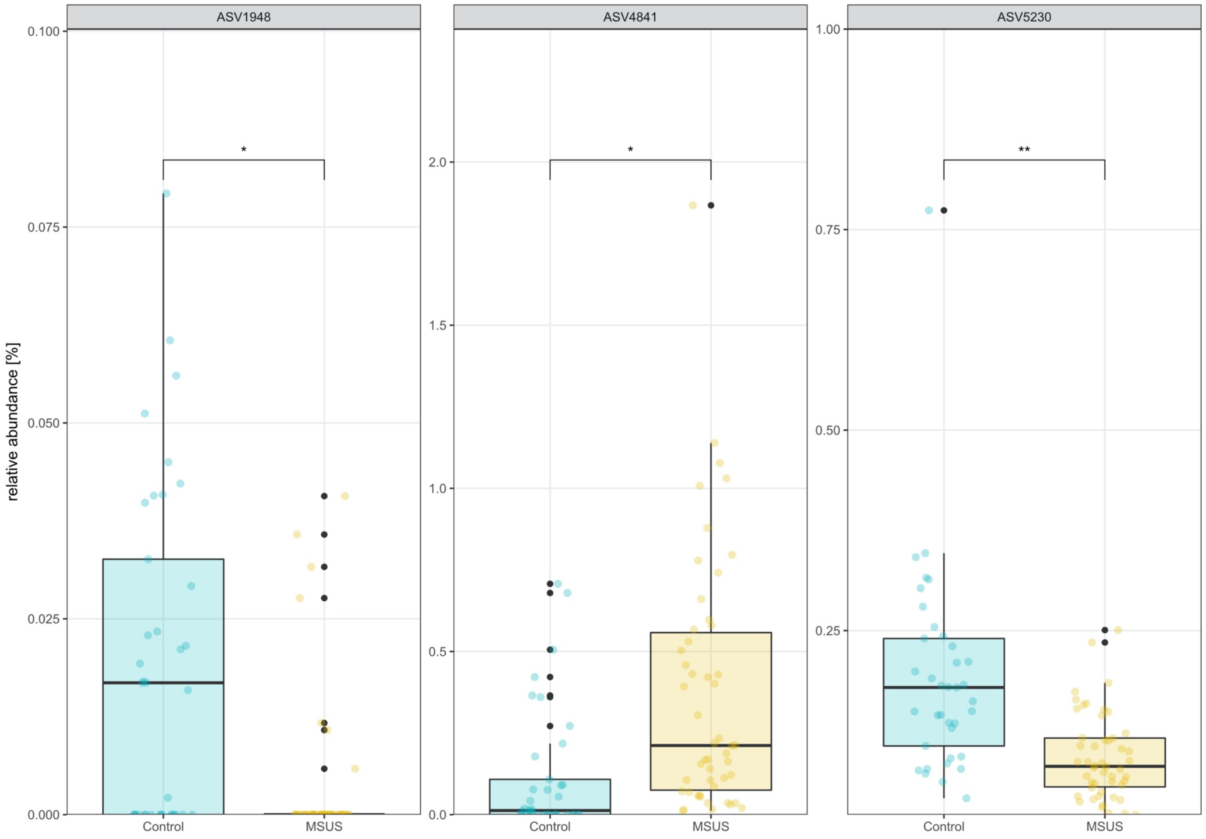

Supplementary Figure 6: Effect of MSUS paradigm on relative abundance of ASVs in 8-week-old F1 mice fecal microbiota. Significantly differentially abundant ASVs between MSUS *versus* control mice are displayed. Boxplot with box elements showing upper and lower quantile and median. Whiskers extend from the hinge to ± 1.5 times the interquartile range or the highest/lowest value. Outliers are indicated as black points, while colored points indicate individual microbiota. Significances were calculated using generalized mixed effect models with FDR correction. * $p < 0.05$; ** $p < 0.01$

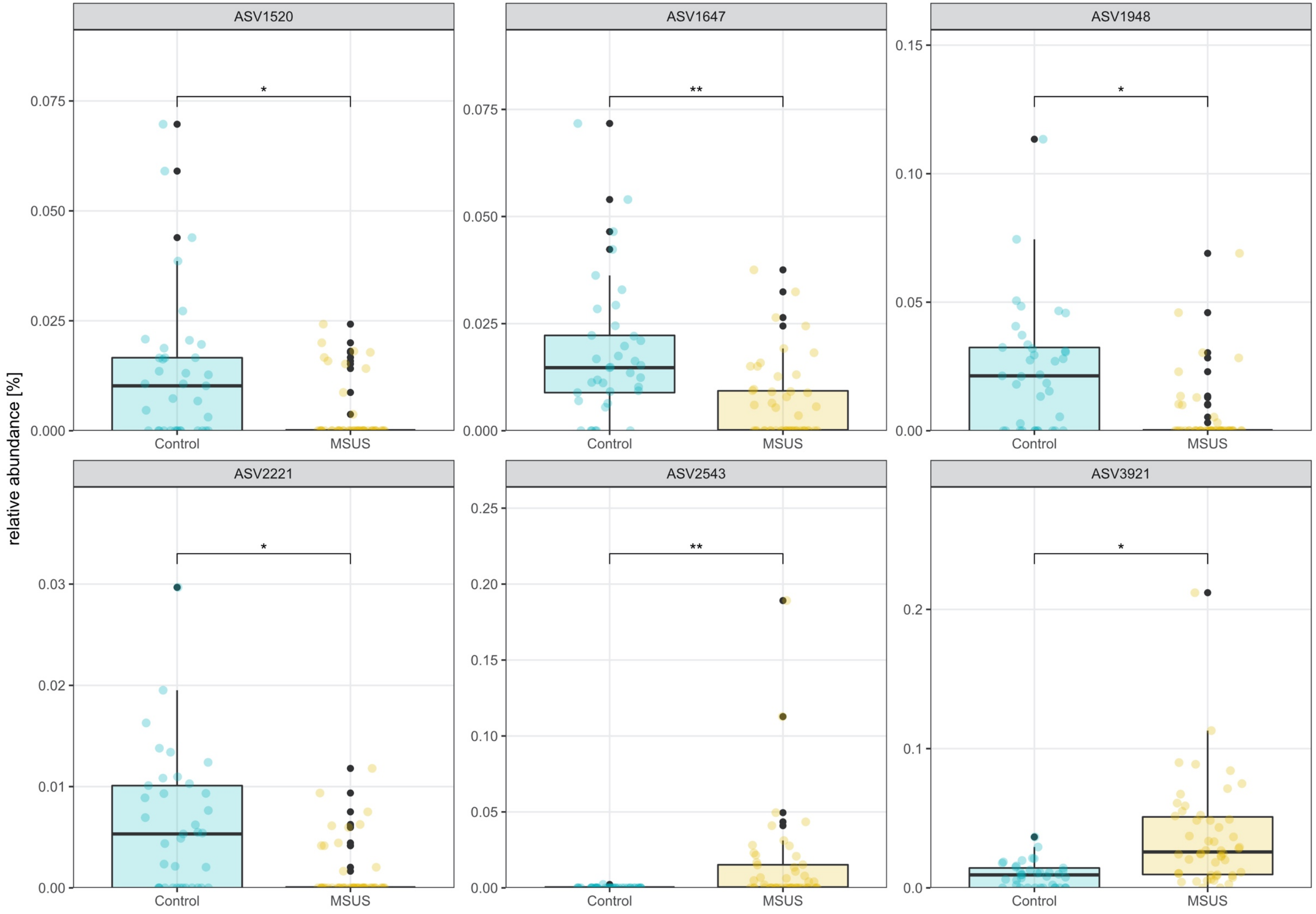

Supplementary Figure 7: Effect of MSUS paradigm on relative abundance of ASVs in 15-week-old F1 mice fecal microbiota. Significantly differentially abundant ASVs between MSUS *versus* control mice are displayed. Boxplot with box elements showing upper and lower quantile and median. Whiskers extend from the hinge to ± 1.5 times the interquartile range or the highest/lowest value. Outliers are indicated as black points, while colored points indicate individual microbiota. Significances were calculated using generalized mixed effect models with FDR correction. * $p < 0.05$; ** $p < 0.01$

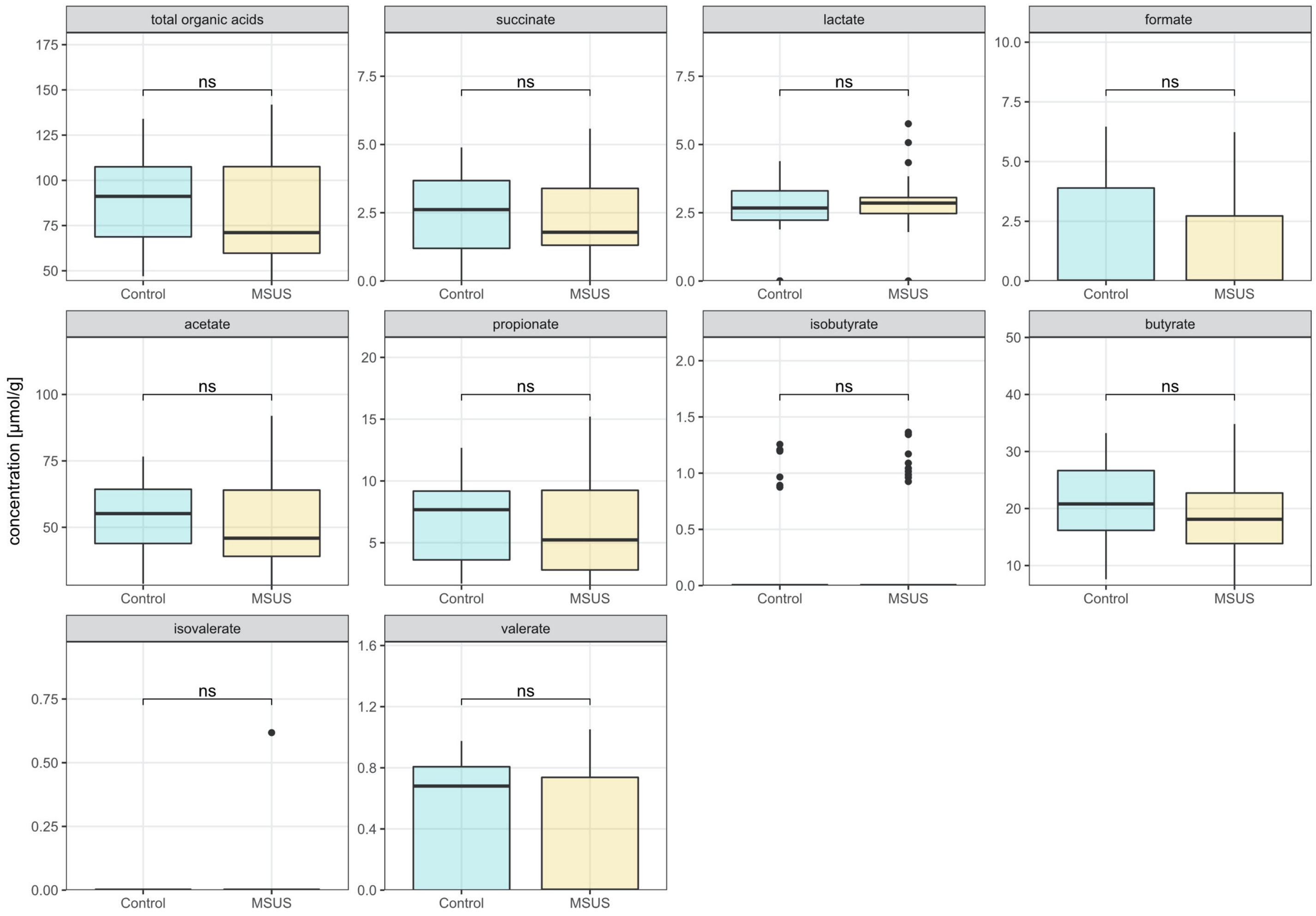

Supplementary Figure 8: Effect of MSUS paradigm on cecal total and specific organic acid concentrations of 30-week-old F1 mice. Comparison of MSUS *versus* control mice is displayed. Metabolite concentration is depicted per gram cecal wet weight. Boxplot with box elements showing upper and lower quantile and median. Whiskers extend from the hinge to ± 1.5 times the interquartile range or the highest/lowest value. Outliers are indicated as black points. Significances were calculated using generalized mixed effect models with FDR correction. ns: not significant

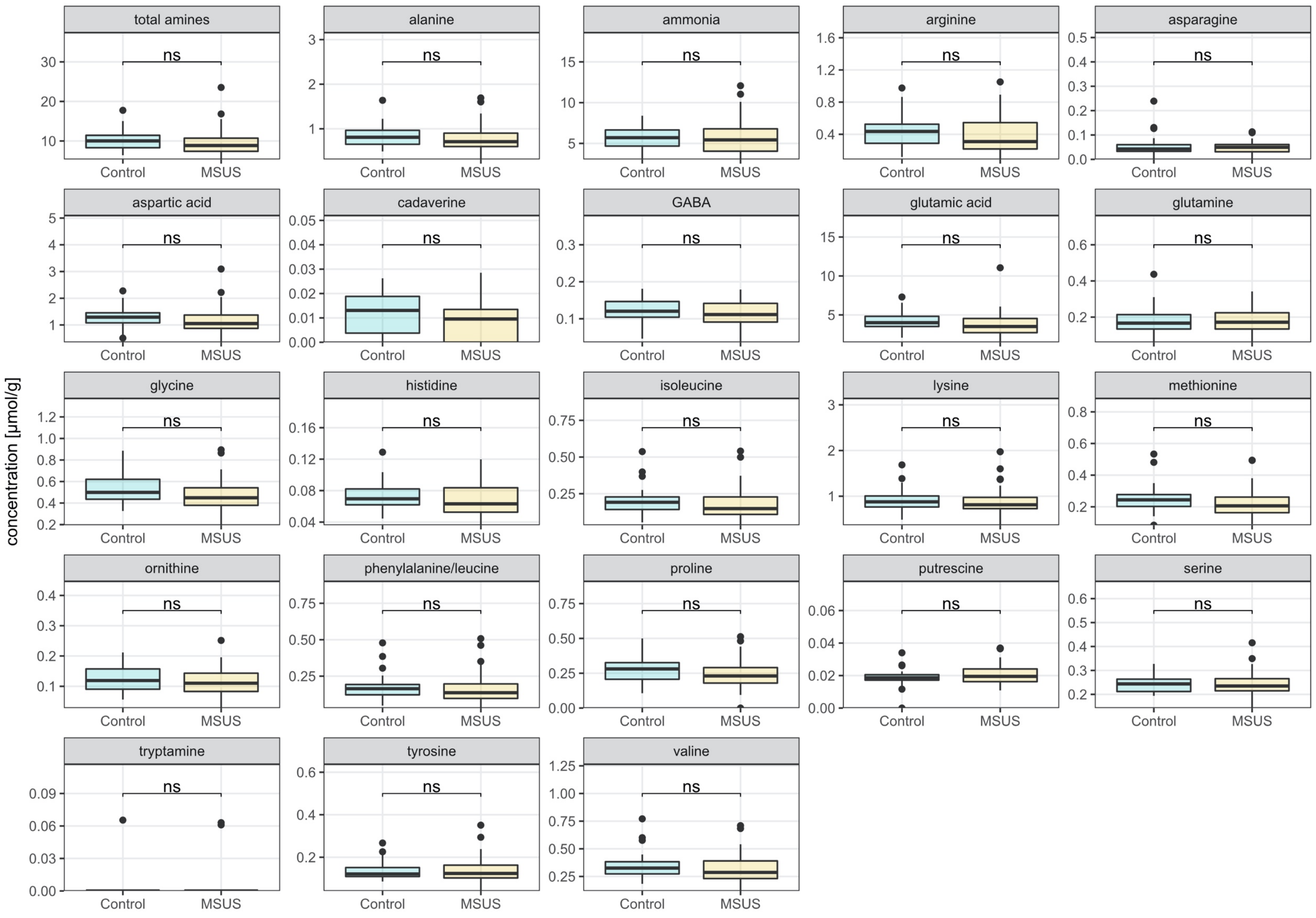

Supplementary Figure 9: Effect of MSUS paradigm on cecal amine, amino acid, and ammonia concentrations of 30-week-old F1 mice. Comparison of MSUS *versus* control mice is displayed. Metabolite concentration is depicted per gram cecal wet weight. Boxplot with box elements showing upper and lower quantile and median. Whiskers extend from the hinge to ± 1.5 times the interquartile range or the highest/lowest value. Outliers are indicated as black points. Significances were calculated using generalized mixed effect models with FDR correction. ns: not significant

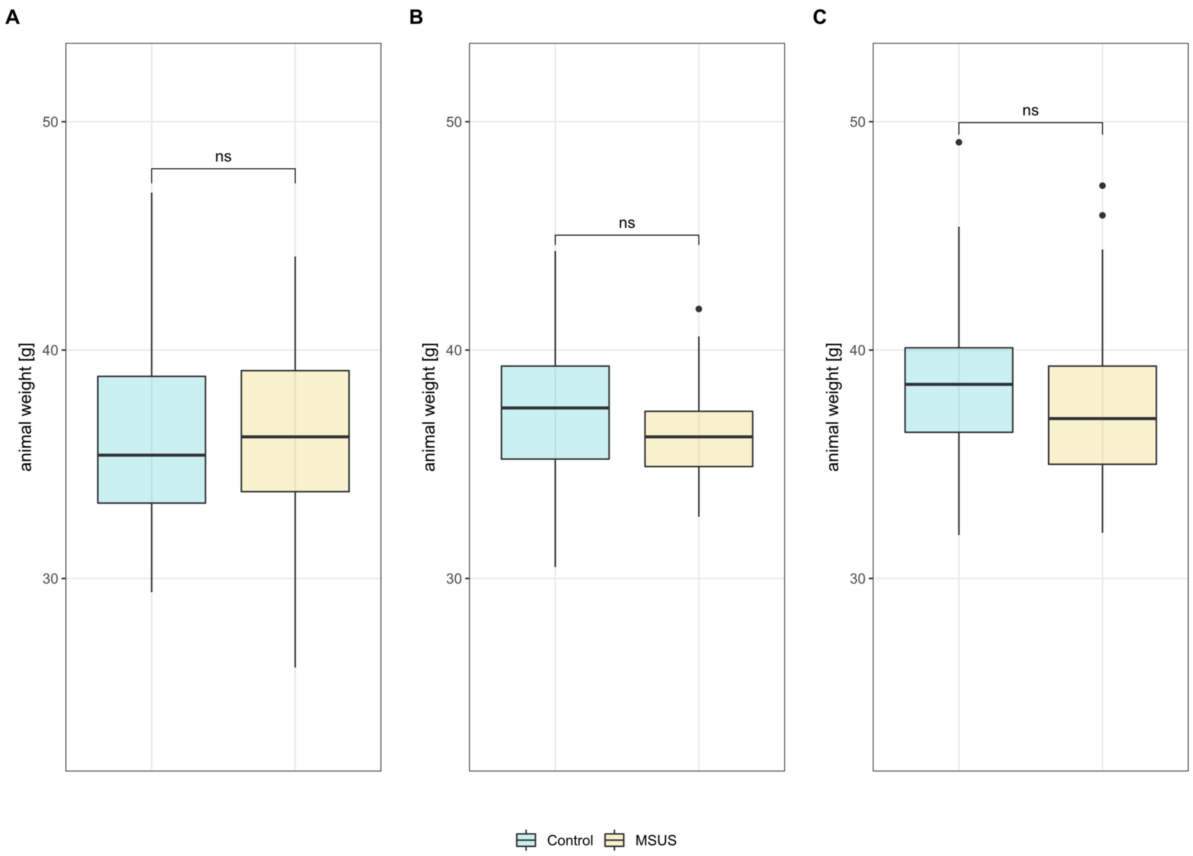

Supplementary Figure 10: Effect of MSUS paradigm on animal weight of F1, F2, and F3 mice. Comparison of MSUS *versus* control mice is displayed. Weight in gram of 30-week-old mice is depicted for (A) F1 and (C) F3, weight of 28-week-old mice is depicted for (B) F2. Boxplot with box elements showing upper and lower quantile and median. Whiskers extend from the hinge to ± 1.5 times the interquartile range or the highest/lowest value. Outliers are indicated as black points. Significances were calculated using generalized mixed effect models with FDR correction. ns: not significant

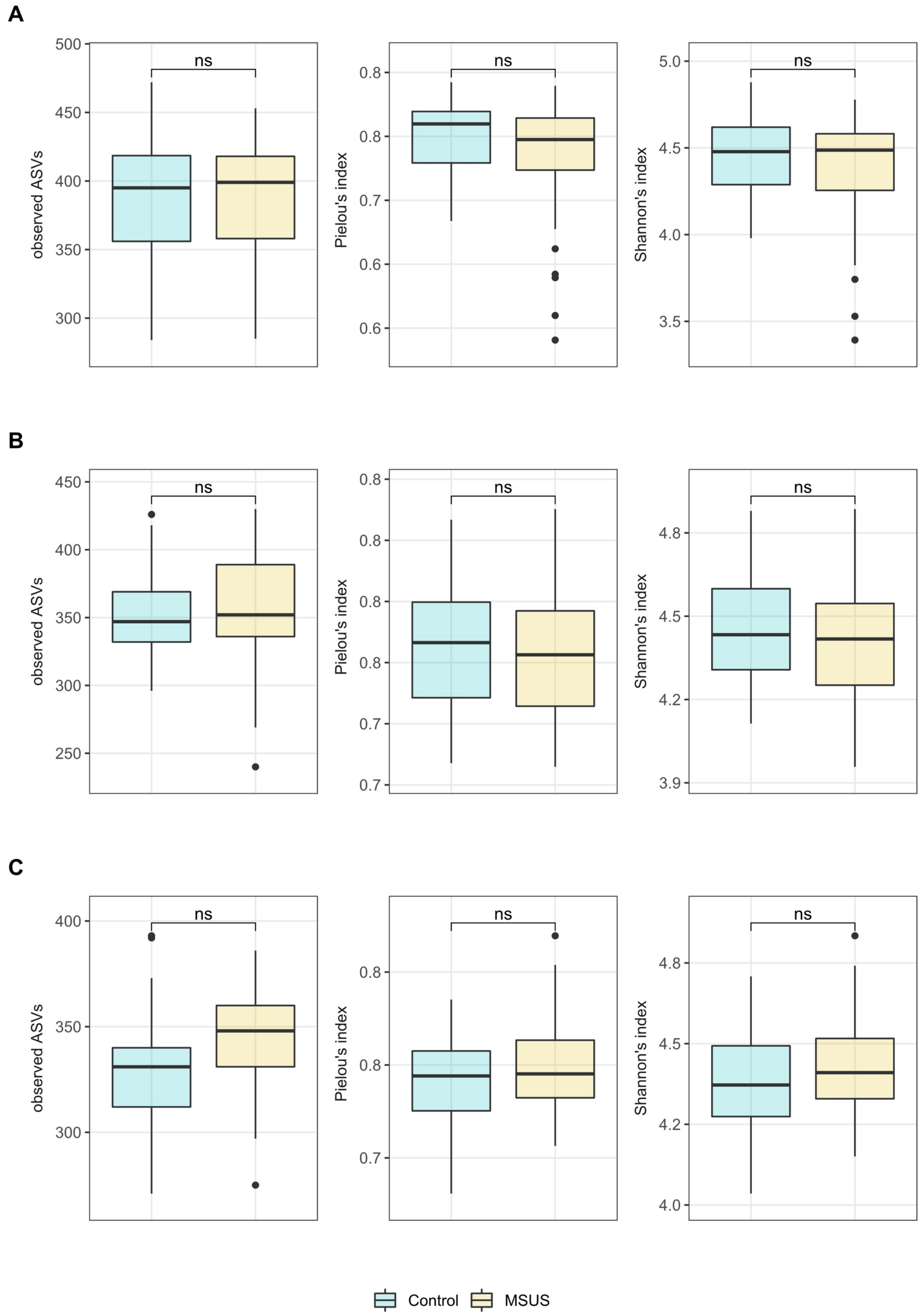

Supplementary Figure 11: Effect of MSUS paradigm on alpha diversity metrics of F1, F2, and F3 mice fecal microbiota. Comparison of richness (observed ASV), evenness (Pielou's index) and Shannon-diversity between MSUS and control is displayed. Alpha diversity of 30-week-old mice is depicted for (A) F1, and (C) F3, and alpha diversity of 28-week-old mice is depicted for (B) F2. Boxplot with box elements showing upper and lower quantile and median. Whiskers extend from the hinge to ± 1.5 times the interquartile range or the highest/lowest value. Outliers are indicated as black points. Significances were calculated using log_2 transformed metrics and generalized mixed effect models with FDR correction. ns: not significant

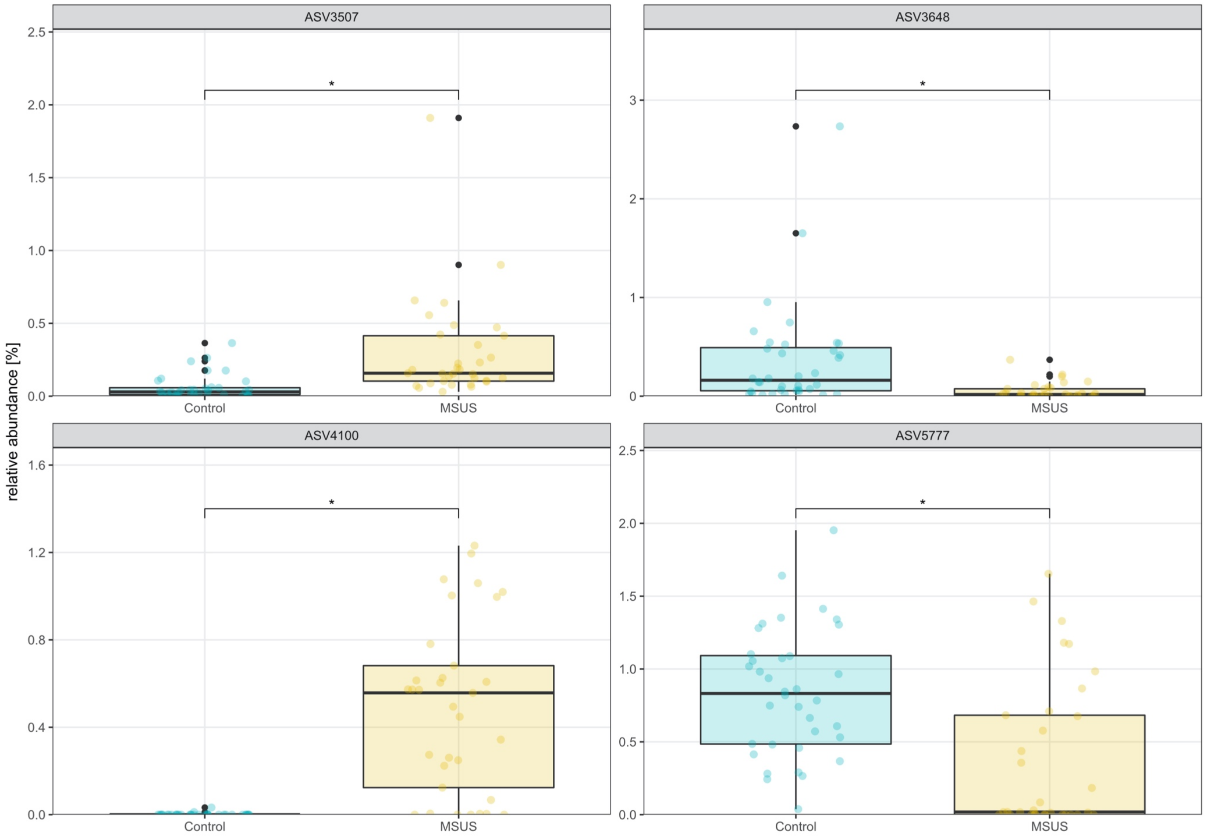

Supplementary Figure 12: Effect of MSUS paradigm on relative abundance of ASVs in 28-week-old F2 mice fecal microbiota. Significantly differentially abundant ASVs between MSUS *versus* control mice are displayed. Boxplot with box elements showing upper and lower quantile and median. Whiskers extend from the hinge to ± 1.5 times the interquartile range or the highest/lowest value. Outliers are indicated as black points, while colored points indicate individual microbiota. Significances were calculated using generalized mixed effect models with FDR correction. * $p < 0.05$

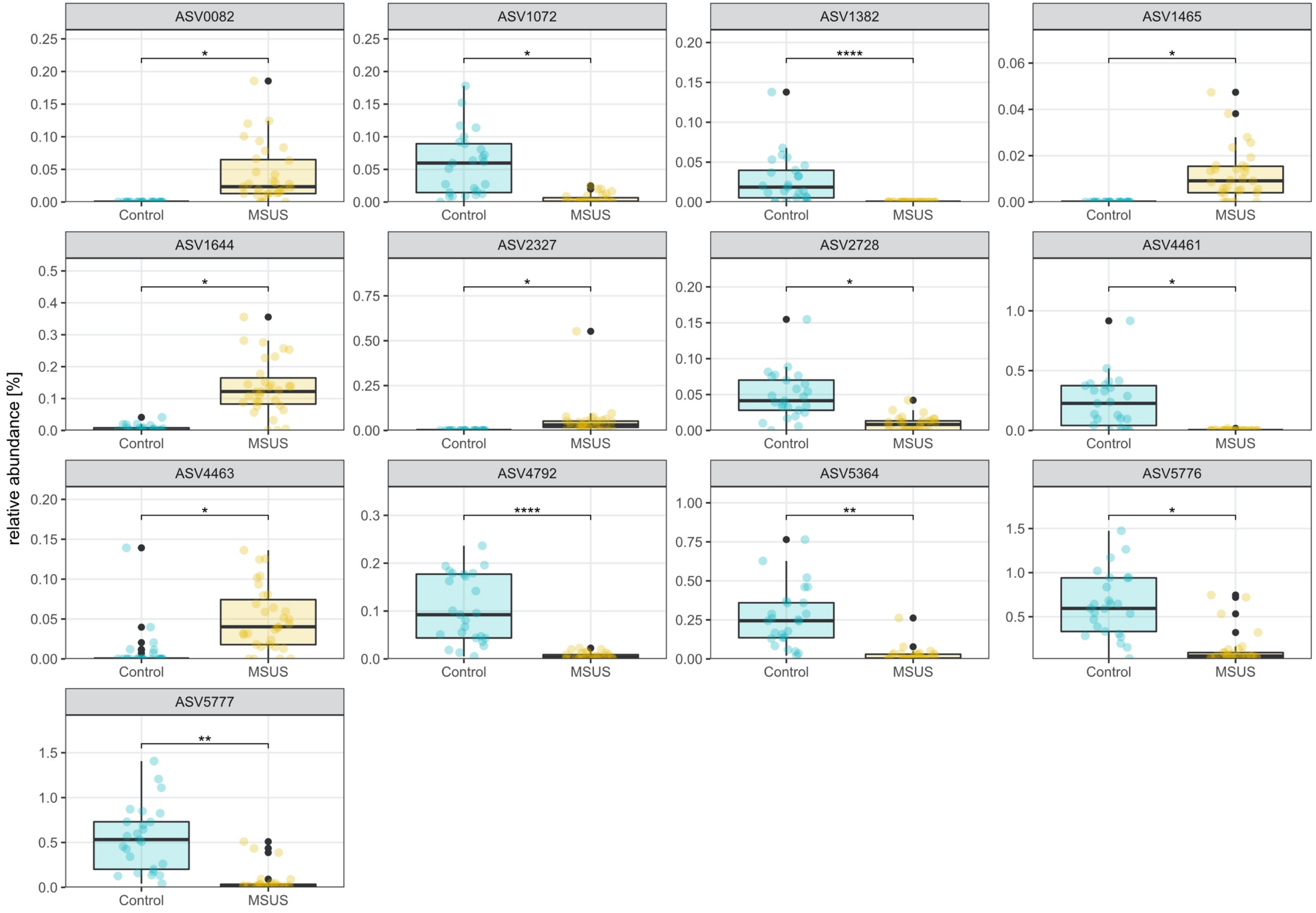

Supplementary Figure 13: Effect of MSUS paradigm on relative abundance of ASVs in 30-week-old F3 mice fecal microbiota. Significantly differentially abundant ASVs between MSUS *versus* control mice are displayed. Boxplot with box elements showing upper and lower quantile and median. Whiskers extend from the hinge to ± 1.5 times the interquartile range or the highest/lowest value. Outliers are indicated as black points, while colored points indicate individual microbiota. Significances were calculated using generalized mixed effect models with FDR correction. * $p < 0.05$; ** $p < 0.01$; **** $p < 0.0001$

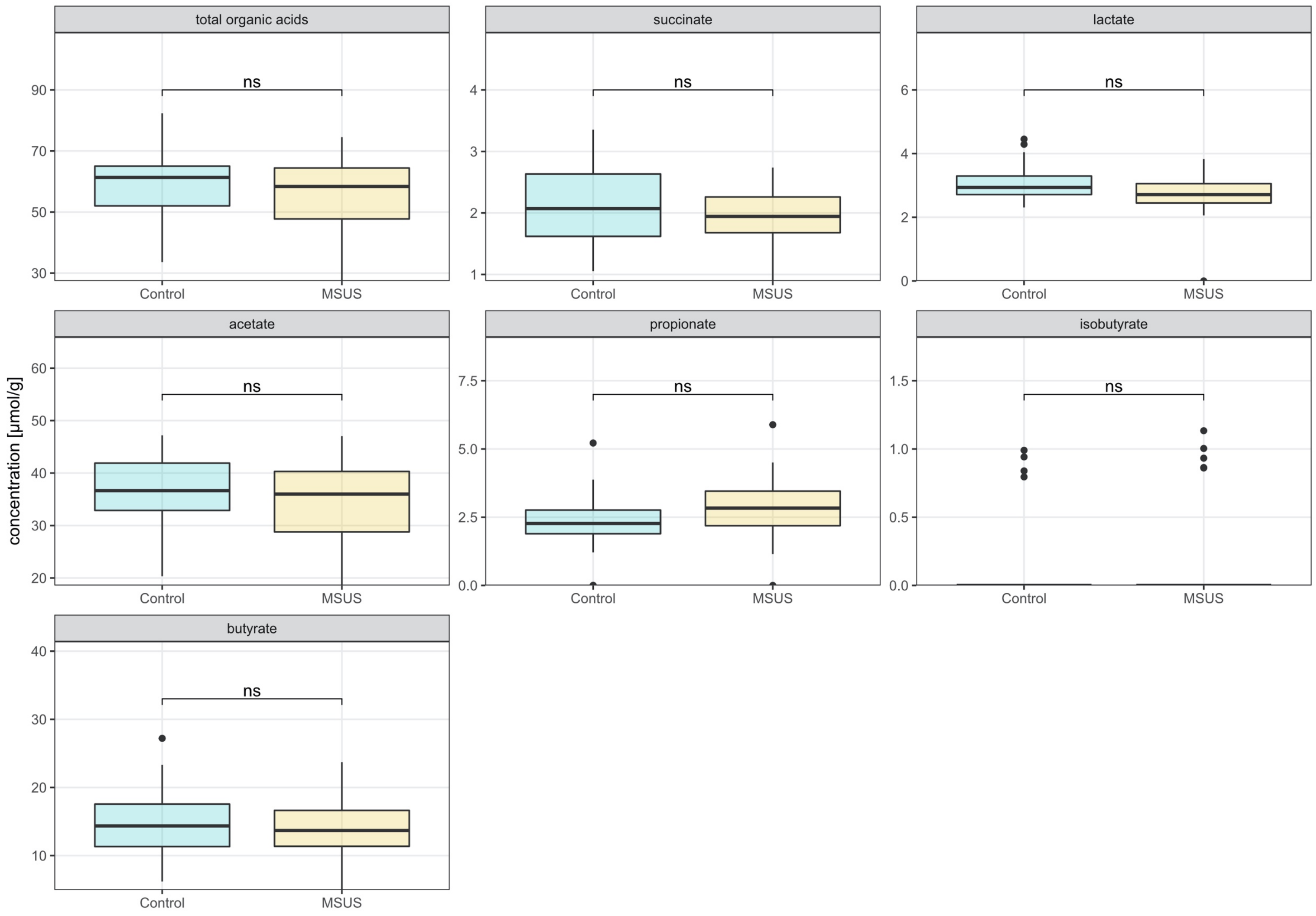

Supplementary Figure 14: Effect of MSUS paradigm on cecal total and specific organic acid concentrations of 28-week-old F2 mice. Comparison of MSUS *versus* control mice is displayed. Metabolite concentration is depicted per gram cecal wet weight. Boxplot with box elements showing upper and lower quantile and median. Whiskers extend from the hinge to ± 1.5 times the interquartile range or the highest/lowest value. Outliers are indicated as black points. Significances were calculated using generalized mixed effect models with FDR correction. ns: not significant

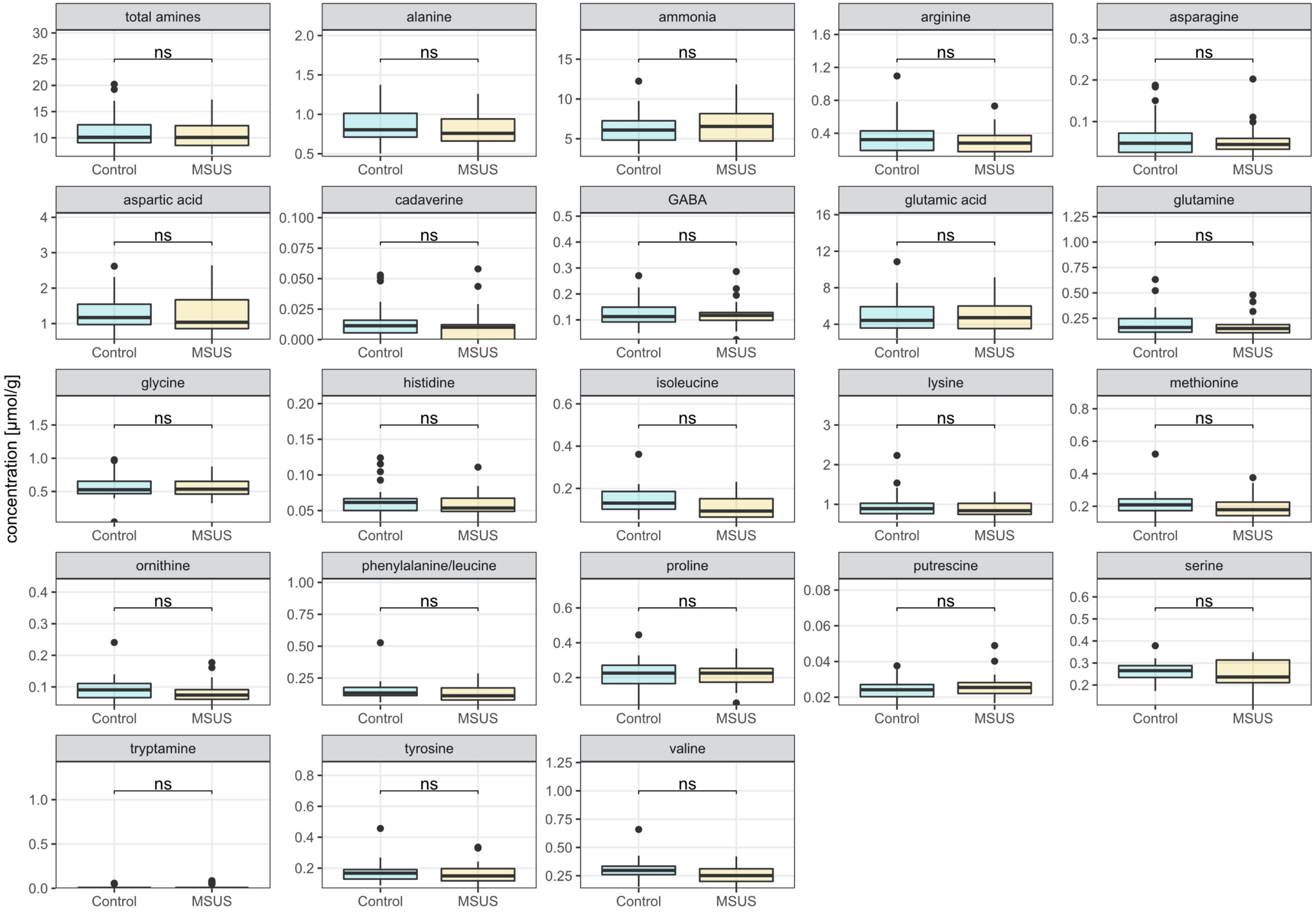

Supplementary Figure 15: Effect of MSUS paradigm on cecal amine, amino acid, and ammonia concentrations of 28-week-old F2 mice. Comparison of MSUS *versus* control mice is displayed. Metabolite concentration is depicted per gram cecal wet weight. Boxplot with box elements showing upper and lower quantile and median. Whiskers extend from the hinge to ± 1.5 times the interquartile range or the highest/lowest value. Outliers are indicated as black points. Significances were calculated using generalized mixed effect models with FDR correction. ns: not significant

Supplementary Table 1: Statistical analysis of pairwise comparison of different beta diversity metrics across life span and between different phenotyping groups of control fecal microbiota. Significances were calculated using PERMANOVA, and permutation test for dispersion, both including FDR correction**.**

metric	comparison	*p*-value*	R^2	dispersion *p*-value*
binary Jaccard index	PND30vsPND22	0.008	0.162	0.665
	WEEK08vsPND22	0.006	0.205	0.825
	WEEK15vsPND22	0.006	0.232	0.471
	WEEK30vsPND22	0.004	0.216	0.665
	WEEK08vsPND30	0.003	0.106	0.086
	WEEK15vsPND30	0.003	0.120	0.011
	WEEK30vsPND30	0.003	0.118	0.055
	WEEK15vsWEEK08	0.045	0.056	0.196
	WEEK30vsWEEK08	0.024	0.057	0.530
	WEEK30vsWEEK15	0.016	0.059	0.825
	GR2vsGR1	0.927	0.104	0.825
weighted Jaccard index	PND30vsPND22	0.008	0.180	0.007
	WEEK08vsPND22	0.006	0.196	0.031
	WEEK15vsPND22	0.006	0.214	0.086
	WEEK30vsPND22	0.004	0.195	0.018
	WEEK08vsPND30	0.003	0.120	0.007
	WEEK15vsPND30	0.003	0.122	0.022
	WEEK30vsPND30	0.003	0.129	0.007
	WEEK15vsWEEK08	0.006	0.064	0.972
	WEEK30vsWEEK08	0.003	0.067	0.665
	WEEK30vsWEEK15	0.003	0.076	0.665
	GR2vsGR1	0.592	0.109	0.972

* FDR-adjusted; GR1: group 1 (behavioral phenotyping before breeding); GR2: group 2 (breeding before behavioral phenotyping); PND: postnatal day

Supplementary Table 2: Statistical analysis of comparison of different beta diversity metrics between F1 MSUS and control fecal microbiota across life span. Significances were calculated using PERMANOVA, and permutation test for dispersion, both including FDR correction**.**

**metric**	**age compared**	***p*-value***	**R^2**	**dispersion *p*-value***
binary Jaccard index	PND22	0.8	0.188	0.964
	PND30	0.002	0.063	0.710
	WEEK08	0.002	0.061	0.964
	WEEK15	0.002	0.067	0.964
	WEEK30	0.002	0.060	0.710
weighted Jaccard index	PND22	0.119	0.209	0.014
	PND30	0.002	0.058	0.870
	WEEK08	0.119	0.052	0.979
	WEEK15	0.075	0.055	0.710
	WEEK30	0.119	0.051	0.964

* FDR-adjusted; PND: post-natal day

Supplementary Table 3: Statistical analysis of comparison of different beta diversity metrics between MSUS and controls of F1, F2, and F3 fecal microbiota. Significances were calculated using PERMANOVA, and permutation test for dispersion, both including FDR correction**.**

**metric**	**age compared**	***p*-value***	R^2	**dispersion *p*-value***
binary Jaccard index	F1 (WEEK30)	0.004	0.060	0.632
	F2 (WEEK28)	0.004	0.077	0.632
	F3 (WEEK30)	0.004	0.131	0.868
weighted Jaccard index	F1 (WEEK30)	0.101	0.051	0.868
	F2 (WEEK28)	0.048	0.071	0.632
	F3 (WEEK30)	0.011	0.099	0.868

* FDR-adjusted

Supplementary Table 4: Time point and number of fecal samples collected for F1, F2, and F3 control and MSUS mice.

generation	treatment	age	mice	cages	litters
F1	control	PND22	11	3	3
	MSUS	PND22	12	3	3
	control	PND30			
		8-weeks-old	37	11	13
		15-weeks-old			
	MSUS	PND30			
		8-weeks-old	50	12	11
		15-weeks-old			
	control	30-weeks-old	35	11	13
	MSUS	30-weeks-old	49	12	11
F2	control	PND21	20	4	4
	MSUS	PND21	25	6	6
	control	16-week-old	36	9	13
	MSUS	16-week-old	36	10	14
	control	28-weeks-old	36	9	14
	MSUS	28-weeks-old	33	9	14
F3	control	30-weeks-old	25	7	8
	MSUS	30-weeks-old	31	8	8

F1: first generation; F2: second generation; F3: third generation; PND: postnatal day
