# Supplementary material for: Transgenerational effects of early life stress on the fecal microbiota in mice
Source: Commun Biol. 2024 May 31;7:670. doi: 10.1038/s42003-024-06279-2 (PMC11143345; doi:10.1038/s42003-024-06279-2)
Supplement: Supplementary file 3 — Description of Additional Supplementary Files [file 42003_2024_6279_MOESM3_ESM.pdf]

Description of Additional Supplementary Files

File name: Supplementary Data 1
Description: Raw data on cecal bacterial metabolite concentrations. Metabolite data is provided as µmol/g.
